# International Normalized Ratio (INR) Sample Rejection in Neck of Femur Fracture Patients: A Retrospective Closed-Loop Study From a Major UK Trauma Centre

**DOI:** 10.7759/cureus.97097

**Published:** 2025-11-17

**Authors:** Mahmoud Mersal, Shady Salama, Mohamed Alsonbaty, Osama Embaby, Abdelrahman Embabi, May Labidi

**Affiliations:** 1 Trauma and Orthopaedics, University Hospitals Birmingham (UHB) Foundation Trust, Birmingham, GBR; 2 Trauma and Orthopaedics, Sandwell and West Birmingham Hospitals NHS Trust, Birmingham, GBR; 3 Emergency Medicine, Royal Surrey Foundation NHS Trust, London, GBR

**Keywords:** international normalized ratio (inr), neck of femur fractures, nof patients, quality improvement, sample rejection, sodium citrate, underfilled tubes

## Abstract

Background

Patients presenting with neck-of-femur (NOF) fractures often require urgent surgery, as prolonged delays beyond 36 hours are associated with increased morbidity, mortality, and length of hospital stay, whereas shorter time-to-surgery intervals have been shown to improve outcomes. Many of these patients are elderly and on anticoagulant therapy; therefore, making accurate International Normalized Ratio (INR) assessment is crucial for determining surgical readiness and anaesthetic safety. The INR reflects the extrinsic pathway of coagulation and is prolonged in patients on warfarin or who have underlying coagulopathies. Inaccurate or rejected INR samples delay operative clearance, prolong fasting, and increase bed occupancy and cost of treatment. A frequent pre-analytical cause of INR rejection is underfilling of sodium citrate tubes, which alters the required 9:1 blood-to-anticoagulant ratio.

Objective

To improve the rejection rate of INR samples through a simple phlebotomy intervention involving staff education and the appropriate use of a discard tube before citrate collection.

Methods

A retrospective two-cycle closed-loop audit was conducted at Heartlands Hospital, part of the University Hospitals Birmingham (UHB) NHS Foundation Trust. The first cycle included NOF fracture patients admitted between July and August 2023, during which 399 INR samples were analysed. Of these, 66 (16.5%) were rejected, 62 (94%) due to underfilling and four (6%) due to haemolysis.

Following targeted interventions, including staff education on correct discard-tube use with butterfly systems and the introduction of shorter-tubing blood collection sets, a second audit cycle was performed and included NOF patients admitted between July and August 2025. In this cycle, 261 INR samples were reviewed, of which 29 (11.1%) were rejected, 27 (93%) for underfilling and two (7%) for haemolysis.

Rejection proportions were compared between cycles, and absolute and relative changes were calculated. Statistical significance of the observed difference was assessed using a two-proportion z-test (two-sided, α = 0.05).

Data collection and review were performed using the UHB trust Prescribing Information and Communication System (PICS) electronic system.

Results

INR sample rejection rate decreased from 16.5% in cycle 1 to 11.1% in cycle 2, an absolute reduction of 5.4% and relative reduction of ~33% (p ≈ 0.11). Among patients taking some form of blood thinner (e.g. warfarin, direct oral anticoagulants, low molecular weight heparin), 132 (33%) in cycle 1 and 56 (25%) in cycle 2, INR rejection occurred in 17.4% and 19.6%, respectively. Most rejections were due to underfilling the INR sample tube.

Conclusions

In trauma patients, particularly those awaiting urgent NOF surgery, preventing INR sample rejection can significantly reduce avoidable operative delays. This closed-loop audit demonstrated that a simple, low-cost intervention focused on correct tube filling, discard-tube use, and appropriate equipment selection led to a clinically meaningful reduction in INR sample rejection rates. Most remaining rejections remain preventable, underscoring the importance of continuous education, reinforcement of best practice, and regular re-audit to sustain long-term improvement.

## Introduction

Neck of femur (NOF) fractures carry substantial morbidity and mortality, particularly among older adults [1]. Time to surgery is a key prognostic factor; delays beyond 36 hours are independently associated with increased complications, prolonged hospital stay, and higher mortality [2,3]. Many of these patients are on anticoagulant therapy, like warfarin, or have underlying coagulopathies. Evaluating coagulation status via the International Normalized Ratio (INR) is therefore essential to safely proceed with anaesthesia and surgical intervention.

INR testing requires blood collection into a 3.2% sodium citrate tube, and maintaining a precise 9:1 blood-to-anticoagulant ratio for accurate results [4]. If the tube is underfilled, excess citrate alters this ratio, leading to falsely prolonged clotting times or laboratory rejection [5,6]. Each blue-top tube is vacuum-calibrated to draw an exact volume; however, when a butterfly collection set is used, air within the tubing may enter the first tube drawn, causing underfilling and disruption of the required ratio. To prevent this pre-analytical error, a discard tube should be filled first to expel air before collecting the sample into the citrate tube, ensuring proper vacuum fill and test validity [7].

In the pre-analytical phase of laboratory medicine, specimen rejection is a well-recognized quality issue. Among coagulation tests, specimen volume error, underfill or overfill, is a leading cause of rejection [8-10]. Because rejected or invalid INR samples require repeat phlebotomy, they delay operative clearance, prolong preoperative fasting, consume staff time, increase bed occupancy, and incur additional cost [11].

In busy trauma services, phlebotomy is often performed under pressure, frequently using winged “butterfly” sets. When a citrate tube is drawn first via a butterfly, any air or dead space in the tubing must be eliminated or primed, commonly via a discard tube, to ensure full vacuum draw. Otherwise, residual air or negative pressure loss may cause underfilling [4,5].

Given the clinical importance of timely surgery in NOF fractures, we conducted a two-cycle closed-loop audit to evaluate the rate of INR sample rejection, implement a targeted phlebotomy intervention, and subsequently reassess post-intervention outcomes. The intervention involved staff education on correct phlebotomy technique, emphasising the discard-tube step when using butterfly sets, and promoting short-tubing blood collection systems to reduce underfilling. We hypothesised that reinforcing these practices would lead to a measurable reduction in sample rejection rates.

## Materials and methods

Setting and study design

This audit was carried out in the Trauma and Orthopaedics (T&O) Department at Heartlands Hospital, part of the University Hospitals Birmingham (UHB) NHS Foundation Trust. It was designed as a retrospective, two-cycle closed-loop audit comparing pre- and post-intervention data.

The first audit cycle analysed data from July to August 2023 and was registered under CARMS-20674, while the second cycle analysed data from July to August 2025 and was registered under CARMS-21805.

The primary objective was to assess the baseline rates of INR sample rejection, implement a targeted phlebotomy intervention, and subsequently evaluate post-intervention outcomes to determine the extent of improvement.

Population and inclusion criteria

The audit included all patients admitted under Trauma and Orthopaedics with a NOF fragility fracture during the specified audit periods in whom an INR sample was attempted.

Patients for whom INR sampling was not performed (e.g., cancelled tests or missing samples) were excluded to ensure accurate calculation of rejection rates and valid denominators.

Data collection and variables

For each audit cycle, data were retrospectively extracted using the Patient Information and Communication System (PICS), the electronic clinical record system utilised at UHB. The PICS system provided access to patient flowsheets and laboratory results, allowing verification of INR values, identification of rejected samples, and review of the recorded reasons for rejection on the system.

Information was gathered for all NOF fracture admissions during the specified audit periods. The dataset included the total number of INR sampling attempts, the number of samples rejected by the laboratory, and the recorded reason for rejection, categorised as underfilling, haemolysis, or other pre-analytical causes. Additional variables included patient demographics and patients’ use of anticoagulant therapy.

For the purposes of this audit, a total INR attempt was defined as any blood draw in which an INR sample was pursued. A rejected sample was defined as one returned by the laboratory marked “refused” or “rejected” due to pre-analytical factors. An underfilling rejection referred specifically to samples rejected for insufficient volume in the sodium citrate tube, resulting in an incorrect 9:1 blood-to-anticoagulant ratio.

Intervention

Following the findings of the first audit cycle, a series of targeted quality improvement measures were implemented within the T&O department and across relevant phlebotomy teams to address the high rate of INR sample rejection.

**Figure 1:**
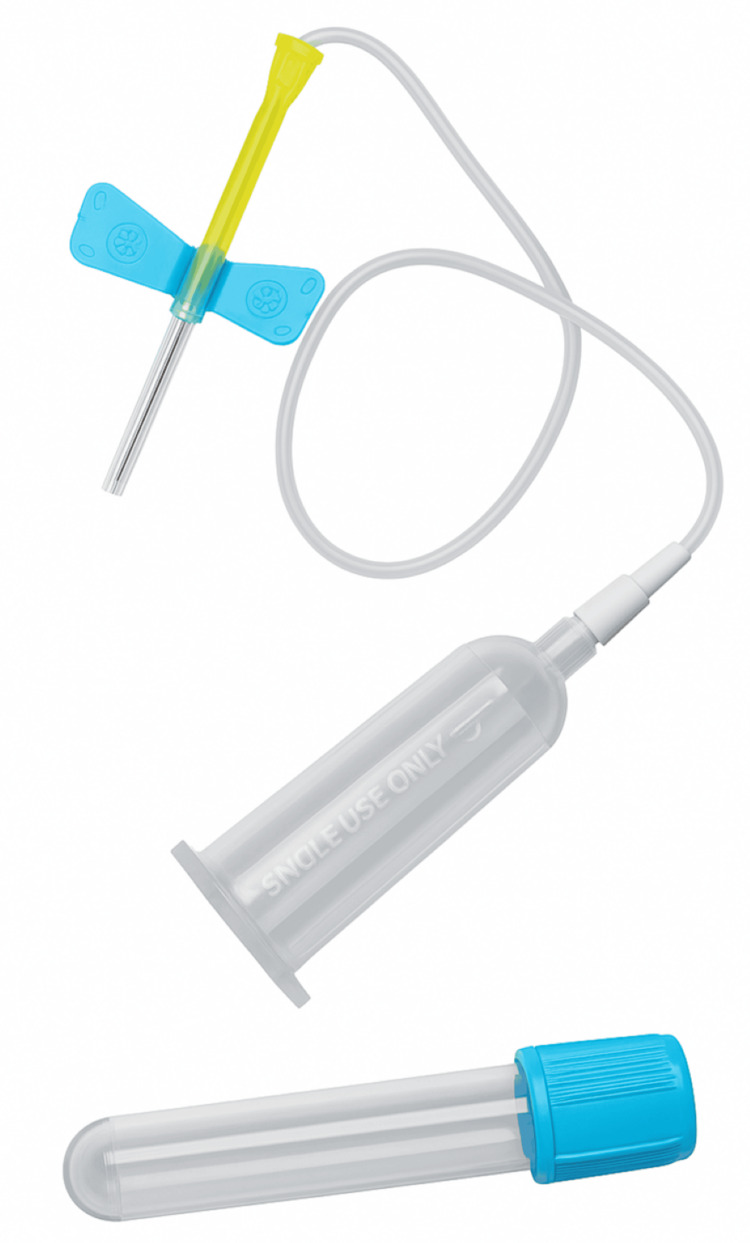
Standard butterfly collection set with long tubing and air space beside a blue-top International Normalized Ratio (INR) sample tube Photograph taken and edited by the authors.

First, a focused educational session was delivered to junior doctors, phlebotomy staff, and ward nursing teams. The sessions reinforced the correct technique for citrate tube sampling, emphasising that when using butterfly or long-tubing collection systems (Figure 1), a discard tube must be drawn first to eliminate residual air from the tubing and ensure complete filling of the subsequent blue-top citrate tube.

**Figure 2:**
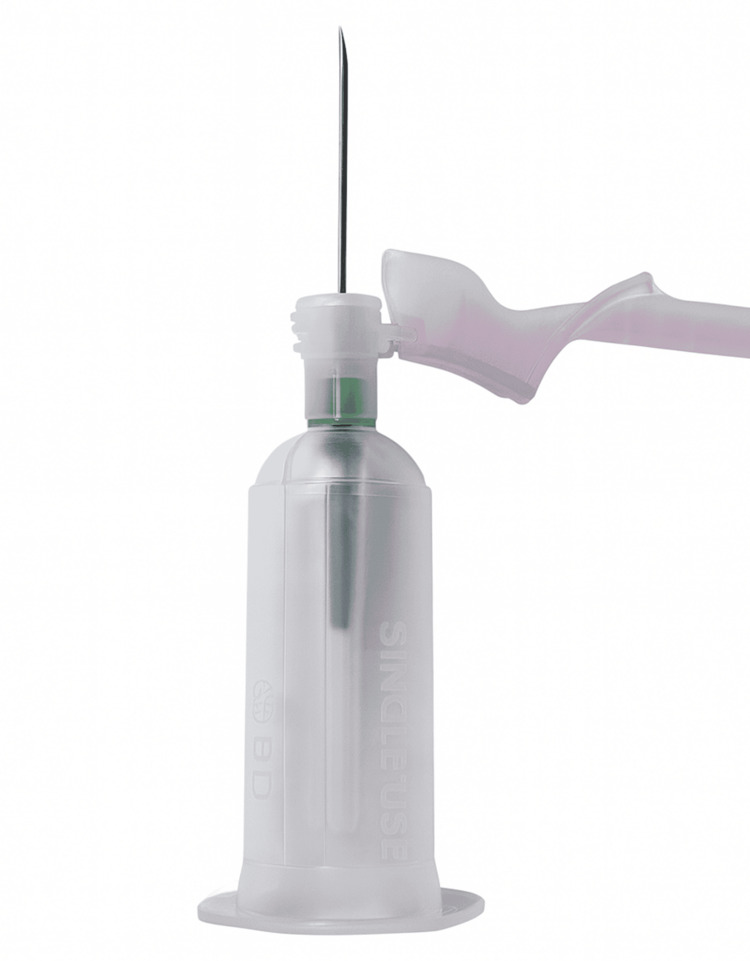
Short-tubing needle system. Photograph taken and edited by the authors.

Second, the department encouraged the adoption and routine stocking of short-tubing blood collection devices, such as the Unistik Vacuflip or equivalent (Figure 2), to reduce dead-space volume and the risk of underfilling.

Finally, visual reminder prompts were placed prominently at all blood sampling points, reinforcing best practice through clear messaging: “Blue top = fill to line, draw discard first if using butterfly.”

These low-cost, easily implementable interventions aimed to standardise technique, minimise pre-analytical error, and ultimately reduce INR sample rejection rates across the service.

Statistical analysis

For each audit cycle, the rejection rate was calculated as the number of rejected samples divided by total INR attempts. Absolute and relative differences between cycles were determined, and 95% confidence intervals (CIs) were calculated. A two-proportion z-test (two-sided, α = 0.05) assessed the statistical significance of change in rejection rates. In addition to a subgroup analysis to evaluate rejection rates among patients receiving anticoagulant therapy.

## Results

During the first audit cycle, a total of 399 INR samples were analysed. The mean patient age was 63.7 years (SD 23.0) and the median age was 68.4 years. The gender distribution included 257 females (64%) and 142 males (36%).

During the second audit cycle, a total of 261 INR samples were reviewed. The mean age was 78.3 years (SD 16.8) and the median age was 82.5 years. The gender distribution included 146 females (67%) and 71 males (33%).

Rejection rates and comparative analysis

The INR sample rejection rate decreased from 16.5% (66/399, 95% CI 13.2 -20.5%) in the first audit cycle to 11.1% (29/261, 95% CI 7.8 - 15.5%) in the second audit cycle, representing a 5.4 percentage-point absolute reduction and a relative reduction of approximately 33%. The two-proportion z-test demonstrated a downward trend that did not reach statistical significance (z = -1.55, p ≈ 0.11).

Among patients on anticoagulant therapy, 132 (33%) in the first audit cycle and 56 (25%) in the second audit cycle were identified. Within this subgroup, INR rejection rates were 17.4% and 19.6%, respectively.

Root cause analysis

Across both audit cycles (Table 1), underfilling of citrate tubes remained the dominant cause of sample rejection. In the first audit cycle, 62 of 66 rejected samples (94%) were due to underfilling, while the remaining four were attributed to haemolysis. Similarly, in the second audit cycle, 27 of 29 rejections (93%) were caused by underfilling and two (7%) by haemolysis.

**Table 1:** Comparative Summary for Both Audit Cycles n (%) = number (percentage).

Parameter	Cycle 1 (Jul–Aug 2023)	Cycle 2 (Jul–Aug 2025)	Change (Δ)
Total INR samples analysed	399	261	—
Mean age (years)	63.7 ± 23.0	78.3 ± 16.8	—
Median age (years)	68.4	82.5	—
Female n (%)	257 (64 %)	146 (67 %)	—
Male n (%)	142 (36 %)	71 (33 %)	—
Rejected samples n (%)	66 (16.5 %)	29 (11.1 %)	–5.4 pp (≈ 33 % ↓)
Patients on anticoagulants n (%)	132 (33 %)	56 (25 %)	—
Rejection on patients taking anticoagulants n (%)	23 (17.4%)	11 (19.6%)	—
Underfilling n (%)	62 (94 %)	27 (93 %)	—
Haemolysis n (%)	4 (6 %)	2 (7 %)	—
Two-proportion z-test (p)	—	—	p = 0.11 (NS)

This consistency indicates a persistent pre-analytical problem primarily linked to sampling technique rather than patient or laboratory factors. Although the observed improvement was not statistically significant, the reduction in rejection rate is operationally meaningful, reflecting enhanced awareness, fewer repeat venepunctures, and improved workflow efficiency.

## Discussion

Main findings

In this audit of NOF fracture patients awaiting surgery, our targeted intervention, reinforcing the use of a discard tube with butterfly-based venepuncture and encouraging shorter-tubing collection systems, coincided with a reduction in INR sample rejection from 16.5% to 11.1%, corresponding to a 33% relative drop. While this fall did not achieve statistical significance (p = 0.11), the direction and magnitude are operationally meaningful in a high-throughput clinical environment. The persistence of underfilling as the dominant cause of rejection highlights that the intervention tackled a major contributor but did not eliminate the problem entirely.

Interpretation in context

Pre-analytical error accounts for a large share of laboratory mistakes, especially in coagulation assays. A review on haemostasis testing noted that lack of standardisation and collection issues, such as wrong tube fill volume, are among the most frequent error sources in coagulation laboratories [5]. In general laboratory studies, “insufficient specimen volume” ranks high among rejection causes, often representing the largest single category of pre-analytical failures [12]. Moreover, specific to coagulation testing, case series have documented that improper blood-to-anticoagulant ratio (i.e. underfilling) is a recurring error leading to sample invalidation or rejection [12].

In orthopaedic settings, especially among hip-fracture (NOF) patients, preoperative coagulation status is critical for both surgical timing and anaesthetic safety. Delays in surgery are independently associated with increased mortality and morbidity. For instance, a large cohort study of hip-fracture patients found that waiting beyond 24 hours was associated with higher 30-day mortality (6.5% vs 5.8%, adjusted) [3]. A meta-analysis reviewing timing of surgery supports similar conclusions; delays beyond 48 hours are associated with worse outcomes [13]. Therefore, reducing avoidable delays, e.g., from repeat sampling, is not just a laboratory goal but a clinical necessity.

While some laboratory systems may tolerate slight underfill volumes, for example ≥ 70% of tube capacity, under validated reagents, most institutions maintain strict rejection thresholds to preserve standardisation and avoid analytic bias [6]. In our setting, enforced rejection of underfilled tubes is safer from a medico-legal and quality-control standpoint, even if marginal underfills might occasionally pass analytic muster.

Strengths and innovations

One strength of this study is its focus on a clinically relevant patient subgroup, NOF fracture patients, where avoiding delay is crucial. The matched audit periods, same months in different years, help mitigate seasonal or operational bias. Recording reason codes in the second audit cycle allowed a root-cause analysis, confirming that underfilling remained the primary failure mode. The intervention was low-cost and feasible: educational reinforcement plus minor equipment adjustments, without capital expenditure. This real-world approach enhances reproducibility in other centres.

Limitations

This study was conducted at a single centre, which may limit the external generalisability of its findings, particularly to laboratories operating with different reagent systems or rejection policies. Although the observed reduction in rejection rates was clinically meaningful, the non-significant p-value suggests limited statistical power, a larger sample size would be required to confirm these results.

Recommendations and sustainability

To consolidate the improvements achieved and promote sustained quality enhancement, several actions are recommended. The discard-tube step should be formally incorporated into standard operating procedures, ward protocols, and new staff induction programmes to ensure consistency in practice. Short-tubing collection sets should remain well-stocked and designated as the default equipment across all wards managing NOF patients to minimise underfilling risk.

Regular spot-check audits, such as random reviews of five blue-top tubes each week, should be conducted, with feedback provided directly to clinical teams to reinforce compliance. Future audit cycles should aim to capture additional metadata, including operator identification, sampling technique, butterfly versus straight needle, tube brand, and time from collection to laboratory processing, to allow deeper analysis of contributory factors.

Finally, collaboration with laboratory colleagues is encouraged to explore whether a validated relaxation of rejection thresholds (for example, accepting tubes filled to 70-80% capacity) could be safely implemented within the local reagent system, balancing analytical accuracy with operational efficiency.

Clinical and operational implications

Reducing INR sample rejection is not just a lab-quality issue; it has direct implications in the NOF care pathway. Fewer repeat phlebotomies reduce patient discomfort, staff burden, and potential delays to theatre. Over time, even small reductions in delay propagate to better throughput, fewer fasting times, reduced bed occupancy, and lower cost. In a busy trauma service operational margins are tight, and any avoidable delay yields cumulative benefit.

## Conclusions

A simple, low-cost intervention, reinforcing discard-tube use and adopting short-tubing collection sets, significantly improved workflow efficiency and reduced INR sample rejection in neck of femur fracture patients. While not statistically significant, this improvement enhances workflow efficiency and patient safety, underscoring the value of continued education, monitoring, and re-audit to sustain long-term quality gains.
